# Evidence against pain specificity in the dorsal posterior insula

**DOI:** 10.12688/f1000research.6833.1

**Published:** 2015-07-24

**Authors:** Karen D. Davis, M. Catherine Bushnell, Gian Domenico Iannetti, Keith St. Lawrence, Robert Coghill

**Affiliations:** 1 Institute of Medical Science and Department of Surgery, University of Toronto, Toronto, ON, M5T 2S8, Canada; 2 Division of Brain Imaging and Behaviour - Systems Neuroscience, Toronto Western Research Institute, Toronto, ON, M5T 2S8, Canada; 3 Joint Department of Medical Imaging, University Health Network, Toronto, ON, M5T 2S8, Canada; 4 Pain and Integrative Neuroscience Branch, Division of Intramural Research, National Center for Complementary and Integrative Health (NCCIH), National Institutes of Health, Bethesda, MD, 20892-1302, USA; 5 Department of Neuroscience, Physiology and Pharmacology, University College London, London, WC1E 6BT, UK; 6 Lawson Health Research Institute, London, ON, N6A 4V2, Canada; 7 Department of Medical Biophysics, The University of Western Ontario, London, ON, N6A 4V2, Canada; 8 Department of Anesthesiology, Cincinnati Children’s Hospital, Cincinnati, OH, 45229-3026, USA

**Keywords:** Pain, insula, brain imaging, ASL

## Abstract

The search for a “pain centre” in the brain has long eluded neuroscientists.  Although many regions of the brain have been shown to respond to painful stimuli, all of these regions also respond to other types of salient stimuli. In a recent paper, Segerdahl *et al.* (Nature Neuroscience, 2015)  claims that the dorsal posterior insula (dpIns) is a pain-specific region based on the observation that the magnitude of regional cerebral blood flow (rCBF) fluctuations in the dpIns correlated with the magnitude of evoked pain.  However, such a conclusion is, simply, not justified by the experimental evidence provided.  Here we discuss three major factors that seriously question this claim.

There are three major factors that we feel negate the claims of the recent study by Segerdahl *et al.* ^1^ that the dorsal posterior insula (dpIns) is a pain-specific area of the brain.

First, the evidence that the dpIns is specific is lacking based on the experimental design and data analysis employed. The methodological approach used by Segerdahl *et al.* ^1^ was to induce an ongoing pain with capsaicin and then to correlate pain intensity ratings with brain perfusion changes using arterial spin labeling (ASL). ASL is an MRI-based perfusion method that can measure fluctuations in rCBF (akin to PET imaging) without the need for a stimulus, and so its application to study ongoing pain is promising. ASL has been previously used by others ^2, 3^ to identify acute and chronic pain-related changes in regional cerebral blood flow (rCBF) but the way Segerdahl *et al.* ^1^ applied it has several shortcomings. The choice of Segerdahl *et al.* ^1^ to collect multi-delay ASL data resulted in rCBF images sampled at infrequent intervals of ~45s, which represents a statistically challenging condition because of the small number of data collected. The control experiment using vibrotactile stimuli comprised a very short scan with even fewer data points in only seven subjects – a design that did not match the already low statistical power of the capsaicin experiment. Therefore, the analysis was underpowered and does not constitute a valid control for the pain experiment. This likely contributed to the minimal activation detected anywhere in the brain during the vibrotactile stimulation. The skin is richly innervated by rapidly adapting, low-threshold mechanoreceptors, so this absence of activation is of substantial concern. Even very early PET studies of regional cerebral blood flow (CBF) found robust vibrotactile activation of primary and secondary somatosensory cortex (S1, S2), and the adjacent posterior insula ^4, 5^. Most importantly, unlike previous investigations where CBF was directly and statistically compared between pain and innocuous stimulation to evaluate specificity of activation ^5, 6^, the Segerdahl *et al.* ^1^ study performed no such key statistical comparison. Without this direct comparison, and in the absence of a control for vibration intensity, or for stimulus saliency, claims of specificity and pain intensity coding simply cannot be made ^7^. This comparison is crucial given the evidence of a vast predominance of low threshold mechanoreceptive neurons in the posterior insula ^8^ and robust vibrotactile activation of the insula (e.g., see 4).

Second, the proposition of a very specific “spot” dedicated to pain is critically dependent on the ability of the methodology to localize findings precisely. However, it is challenging to derive an accurate, group-averaged localization of activation within the dpIns given 1) the large intersubject anatomical variability of the insula, in particular the posterior gyri ^9^ and 2) the method of realignment and morphing of brain anatomy into a common space to produce group maps. Inspection of the reported dpIns peak coordinate in the Juelich histologic atlas reveals that this peak activation has a 63% probability of being in the parietal operculum (S2, OP2), and only a 31% probability of being in the insular cortex. These areas are in close approximation, but S2 has a well-documented involvement in both nociceptive and innocuous somatosensory processing (e.g., see 8). No additional procedures were performed to functionally distinguish these two regions.

Third, the interpretation of the findings and proposition of a specific pain center was made without taking into consideration a large body of scientific evidence addressing the brain mechanisms that contribute to pain. Theories of pain have been debated for centuries ^10^, and we still do not know how pain is represented in the brain despite decades of searching for a pain specific brain center. This pursuit for a simple, single pain center however is no longer necessary given the enormity of human neuroimaging data indicating that there is no such dedicated center. Each and every brain area that contains nociceptive neurons also contains non-nociceptive neurons, and neuroimaging has shown that each brain area that responds to noxious stimuli can also respond to non-noxious stimuli ^11^. Rather, multiple, converging lines of evidence strongly indicate that the experience of pain - as any other conscious experience - is constructed from highly distributed cortical processes ^5, 12^. For example, many brain regions exhibit activity related to pain intensity (e.g., 12, 13). Furthermore, there are several clinical cases of preserved pain perception despite lesions of critical regions including the insula, anterior cingulate, and even the entire contralateral hemisphere ^14, 15^. Other studies have shown that interactions among multiple brain regions are critical for distinguishing a state of pain from other highly salient events ^16^.

It is also useful to place the findings of Segerdahl *et al.* ^1^ in context given the historical view of insular function. Morphological, physiological and imaging studies throughout the 1980s and 1990s, divided the insula into anterior agranular and posterior granular subregions, with pain-related function attributed to the anterior part, and a variety of other functions, including tactile recognition, attributed to the posterior part (e.g., see 8). Since that time, the anterior insula has been established to be part of a non-specific network related to attention and salience. In addition, there is anatomical and electrophysiological evidence for thermoreceptive processing in the dpIns via a spinal cord lamina 1 pathway ^17^. Although neuroimaging has shown that the dpIns likely has a role in pain and intensity coding, it is critical to reiterate that intensity-coding has also been found for non-pain modalities in this region, including C-fiber mediated pleasant-touch ^18– 20^. The last decades have seen several theories of insula function being put forward ^21^. This balanced view of potential dpIns functions is surprisingly absent from the discussion of Segerdahl *et al.* ^1^. One important theory to consider, put forth by Apkarian’s group ^13^, is that of the “how much” general magnitude-detector function of the insula. Another important theory developed by Craig and colleagues ^17^, proposes the dpIns to be a center for interoceptive integration and awareness. Thus, there are several important issues ^22^ that need to be considered to fully interpret the findings of Segerdahl *et al.* ^1^. One assumption that drove the approach taken was that of the critical role of intensity-coding as being central to finding a “pain specific” center. We challenge this because although intensity certainly is one classic dimension of pain, there are many other dimensions including location, quality, and unpleasantness that together comprise the experience of pain. Furthermore, none of these dimensions are actually required for a fundamental feeling of pain (see the recent theory put forth by Davis *et al.* ^23^).

In conclusion, the extensive evidence about the role of the dpIns is not considered by Segerdahl *et al.* ^1^ and we note that they do not refute this evidence in their claim to have identified a novel, specific pain center in the dpIns. Such simplistic notions of a specific pain center are incorrect, and therefore dangerous at both an intellectual as well as a clinical level. Here, we suggest an alternate concept of the function of the dpIns based on previous theories and a large body of data that strongly indicate that the dpIns likely is involved in pain but overall is a non-specific perceptual way-station, rather than a specific pain centre. Failure to recognize that many regions activated during nociceptive stimulation are engaging in computational processes related to many things other than pain, lead to interpretations that are fraught with reverse inference ^11^, and they encourage neurosurgeons to pursue lesions for pain control, an approach that has largely been shown to be ineffective since the 1960's ^24^. Their promotion of the concept of a single spot in the brain for pain is even more surprising given the enormous amount of data emerging over the last decade showing the representation of brain function in functional networks, rather than “spots” and the newer view of a “dynamic pain connectome” ^25^. Implications of their concept are far-reaching – from basic theories of pain, to development of “pain-o-meter” type diagnostic tests, to establishing a therapeutic target for clinical pain management ^26, 27^.
