# Supplementary material for: Evidence against pain specificity in the dorsal posterior insula
Source: F1000Res. 2015 Jul 24;4:362. [Version 1] doi: 10.12688/f1000research.6833.1 (PMC4566284; doi:10.12688/f1000research.6833.1)
Supplement: Peer review history

# Peer review: Referee response for version 1

**DOI:** 10.5256/f1000research.7347.r9627

**Published:** 2015-09-07

**Authors:** Apkar Vania Apkarian

**Affiliations:** 1 Department of Surgery and Anesthesia, Northwestern University, Chicago, IL, USA

The correspondence by K. Davis and colleagues regarding the recently published study by Segerdahl *et al*. raises important and valid concerns regarding the conclusion of the paper. The issue is important as the report appeared in a high visibility journal, and the authors make the strong claim that they have identified a single "pain center" in the cortex. The latter has been a quest sought by many pain researchers since the advent of neuroimaging technology. Most importantly a lack of adequate statistical power and a proper control are the most obvious technical weaknesses pinpointed by Davis *et al*.. Perhaps it would be informative to elaborate on this issue, specifically regarding how an underpowered study can lead into discovering a brain "specific center" for pain perception, the validity of which is doubted by senior scientists in the field. 

Neuroimaging studies, whether based on BOLD or ASL, when attempting to identify brain activity relative to a task commonly first identify in each participant brain activity related to the task, average these patterns across all participants, and then use a set of statistical criteria to determine what brain areas are statistically significantly conveying information about the task. In the present study only one brain region passed the specific criteria used and thus we have a single brain area related to the task. Not surprisingly the area is the posterior insula. A brain region that 10 years ago was described to be most commonly observed activated area to any painful stimuli ^1^ and currently in a PubMed term-based meta-analysis, neurosynth ( www.neurosynth.org), it is identified (together with the secondary somatosensory cortex) as the region with highest reverse inference probability (z-score > 13.0) for association with the term “pain”, based on 420 publications. Thus, there is good evidence for this region being involved in pain related studies, and in an underpowered study where high thresholds becomes necessary to identify brain activity it is not surprising that only this one region is identified. Additionally one fully expects that with increased power most of the extended set of brain regions identified in neurosynth, whether called ‘neuromatrix’ or ‘pain connectome’, would also be observed independent of the neuroimaging technology used (tip of the iceberg phenomenon). A simple analogy can be derived from astronomy. Modern telescopes provide us with a picture of the sky full of millions of stars and galaxies. However, even today if we look at the sky by a telescope manufactured by Galileo, or having an equivalent resolution, we will still only observe the handful of stars that Galileo was describing 450 years ago.

The other important issue of the paper by Segerdahl *et al*. regards the conceptual implications, an issue that Davis *et al*. mention and again is important to elaborate. In the effort of proving that pain is in and of itself a unique sensory system, a large number of pain scientists have espoused the notion of dedicated real estate in the cortex for pain. Yet, isolation of such a single region has the strong implication that the conscious, subjective, and affective perception of pain is all captured in this one brain area. The latter implies the thought experiment of excising the region and placing it in a dish (perhaps also keeping all the tissue that connects it to the periphery), with which act we can claim to recapitulate pain consciousness in a dish, which seems absurd and inconsistent with modern theories relating the brain to perception ^2^.

Borsook’s commentary on the problems associated with Segerdahl *et al*. publication is also very astute ^3^. He points that the competition to publish in high end journals pushes the scientist into making more extravagant conclusions than even the author herself or himself actually does not trust. Yet ultimately responsibility rests on the peer review process, and the latter is not guaranteed to be full proof.

I have read this submission. I believe that I have an appropriate level of expertise to confirm that it is of an acceptable scientific standard.

## References

[1] ApkarianAVBushnellMCTreedeRDZubietaJK: Human brain mechanisms of pain perception and regulation in health and disease. *Eur J Pain*.2005;9(4) : 10.1016/j.ejpain.2004.11.001463-484 10.1016/j.ejpain.2004.11.001 Reference source 15979027

[2] BalikiMNApkarianAV: Nociception, Pain, Negative Moods, and Behavior Selection. *Neuron*.2015;87(3) : 10.1016/j.neuron.2015.06.005474-491 10.1016/j.neuron.2015.06.005 26247858PMC4529956

[3] BorsookD: Referee Report For: Evidence against pain specificity in the dorsal posterior insula [v1; ref status: approved 1, http://f1000r.es/5o3]. *F1000Res*.2015;4: 10.5256/f1000research.7347.r9663362 10.5256/f1000research.7347.r9663 Reference source PMC456628426401267

# Peer review: Referee response for version 1

**DOI:** 10.5256/f1000research.7347.r9663

**Published:** 2015-07-28

**Authors:** David Borsook

**Affiliations:** 1 Department of Radiology, Massachusetts General Hospital, Boston, MA, USA

**When Research Reports may be Painful - Open Discourse should Prevail**

Karen Davis and coauthors, including notable researchers in the pain imaging field, including Robert Coghill and Catherine Bushnell ^1^, argue strongly against the concept reported in Nature Neuroscience by Segerdahl and colleagues ^2^; a paper that has quickly proven to be highly controversial for many in the field.  The Segerdahl report utilizes an MRI technique known as arterial spin labeling (ASL) to measure quantitative cerebral blood flow as a surrogate marker for neuronal activation ^3^, which in this case, was combined with a model of capsaicin application to the skin to induce a hypersensitivity to heat stimuli in healthy patents ^4^^,^^5^ as a surrogate model of allodynia in chronic pain.  

Many of the arguments raised by Davis *et al*. relate to the validity of the Segerdahl report, and are of a technical nature.  Hopefully, these technical problems can be easily addressed (e.g., in future experiments) or challenged by an understanding of the field and its limitations (as Davis *et al* so eloquently do - see First and Second Arguments in their paper). What is still unclear is why Segerdahl and colleagues, seem to have overlooked considerable prior work using ASL in experimental pain ^6^^-^^8^, post-surgical pain ^9^^-^^11^, and chronic pain ^12^^-^^16^. There is also a noticeable lack of consideration for the limitations of both the imaging technique and the experimental pain model ^5^, which itself in healthy volunteers, has some issues relating to reproducibility and its clinical relevance ^4^.

The concept of discovering or defining a pain specific area in pain patients has to be understood in terms of a long history searching for such an area to target with various therapeutic modalities, most notably, neurosurgery has led the charge.  The evaluation of putative pain specific areas in acute pain models probably has little if any bearing on the clinical condition of chronic pain.   More modern concepts of brain-wide integrative processes are now in vogue.  Davis authors use the definition of “ *Pain-Connectome*” ^17^ which while conceptually is not new, adds to the growing literature of Connectomics ^18^, and should help rid the often used and probably not useful concept or term ‘ *pain matrix*’ from use, given the modern understanding of brain networks.

At best, the Segerdahal contribution has raised a vibrant discussion in the field, at is worst it is setting the field back not only because of its purported methodological inaccuracy (as evaluated by Davis *et al.*), lack of acknowledgement of what has come before, perhaps being too enthusiastic about the results and therefore pushing a notion that is unlikely to be true – finding a single brain area that is a pain specific region.  Publications in high impact journals such as *Nature Neuroscience* ( http://www.nature.com/neuro/index.html) carry a great responsibility, since they can (and usually) contribute to a field moving forward, or in a few cases the field becoming ‘stuck’ because of potentially false concepts that then take time for any field to undo. Hopefully this is not one of those issues relating to how high profile papers may occasionally be problematic as previously commented on, for example: “ *How journals like Nature, Cell and Science are damaging science*” ( http://www.theguardian.com/commentisfree/2013/dec/09/how-journals-nature-science-cell-damage-science).  Having the finding being replicated in the context of chronic pain conditions will be interesting to observe; perhaps Segerdahl *et al*., have these in the planning stage.  This is of particular importance since having reproducible data from chronic pain patients may provide important therapeutic opportunities.

What is still to be defined, through a more detailed connectomic understanding, is whether such brain areas may be important through integration of processes such as chronic pain with other brain regions, in remodeling or reconstituting normalization of brain circuits following treatment for chronic pain.  Such a notion could perhaps be the real excitement of where the field is headed. A healthy discourse in science can only lead to further evolution in the field and should be open and honest.  I believe Davis and colleagues have made such a contribution in their review of the Segerdahl paper.

I have read this submission. I believe that I have an appropriate level of expertise to confirm that it is of an acceptable scientific standard.

## References

[1] DavisKDBushnellMCIannettiGSt LawrenceKCoghillR: Evidence against pain specificity in the dorsal posterior insula. v1; ref status: approved 1, http://f1000r.es/5o3. *F1000Res*.2015;4(362) : 10.12688/f1000research.6833.1 10.12688/f1000research.6833.1 Reference source PMC456628426401267

[2] SegerdahlARMezueMOkellTWFarrarJTTraceyI: The dorsal posterior insula subserves a fundamental role in human pain. *Nat Neurosci*.2015;18(4) : 10.1038/nn.3969499-500 10.1038/nn.3969 Reference source 25751532PMC6783299

[3] BuxtonRBFrankLRWongECSiewertBWarachSEdelmanRR: A general kinetic model for quantitative perfusion imaging with arterial spin labeling. *Magn Reson Med*.1998;40(3) : 10.1016/j.mri.2010.03.035919-927 10.1016/j.mri.2010.03.035 Reference source 9727941

[4] CavalloneLFFreyKMontanaMCJoyalJReginaKJPetersenKLGereauRW: Reproducibility of the heat/capsaicin skin sensitization model in healthy volunteers. *J Pain Res.*.2013;Nov 7(6) : 10.2147/JPR.S53437919-927 10.2147/JPR.S53437 Reference source 24232380PMC3827105

[5] DirksJPetersenKLDahlJB: The heat/capsaicin sensitization model: a methodologic study. *J Pain.*.2003;4(3) : 10.1054/jpai.2003.10122-128 10.1054/jpai.2003.10 Reference source 14622709

[6] OwenDGThomasAWPratoFSSt LawrenceKS: Quantification of pain-induced changes in cerebral blood flow by perfusion MRI. *Pain*.2008;136(1-2) : 10.1016/j.pain.2007.06.0285-96 10.1016/j.pain.2007.06.02 Reference source 17716820

[7] FrölichMADeshpandeHNessTDeutschG: Quantitative changes in regional cerebral blood flow induced by cold, heat and ischemic pain: a continuous arterial spin labeling study. *Anesthesiology*.2012;117(4) : 10.1097/ALN.0b013e31826a8a13857-867 10.1097/ALN.0b013e31826a8a13 Reference source 22913924PMC3727169

[8] MalekiNBrawnJBarmettlerGBorsookDBecerraL: Pain response measured with arterial spin labeling. *NMR Biomed*.2013;26(6) : 10.1002/nbm.2911.664-673 10.1002/nbm.2911. Reference source 23319440PMC3634868

[9] HowardMAKrauseKKhawajaNMassatNZelayaFSchumannGHugginsJPVennartWWilliamsSCRentonTF: Beyond patient reported pain: perfusion magnetic resonance imaging demonstrates reproducible cerebral representation of ongoing post-surgical pain. *PLoS One*.2011;6(2) : 10.1371/journal.pone.0017096e17096 10.1371/journal.pone.0017096 Reference source 21373203PMC3044150

[10] HodkinsonDJKrauseKKhawajaNRentonTFHugginsJPVennartWThackerMAMehtaMAZelayaFOWilliamsSCHowardMA: Quantifying the test-retest reliability of cerebral blood flow measurements in a clinical model of on-going post-surgical pain: A study using pseudo-continuous arterial spin labelling. *Neuroimage Clin.*.2013;3: 10.1016/j.nicl.2013.09.004301-310 10.1016/j.nicl.2013.09.004 Reference source 24143296PMC3797555

[11] O'MuircheartaighJMarquandAHodkinsonDJKrauseKKhawajaNRentonTFHugginsJPVennartWWilliamsSCHowardMA: Multivariate decoding of cerebral blood flow measures in a clinical model of on-going postsurgical pain. *Hum Brain Mapp.*.2015;36(2) : 10.1002/hbm.22652633-642 10.1002/hbm.22652 Reference source 25307488PMC4322468

[12] WasanADLoggiaMLChenLQNapadowVKongJGollubRL: Neural correlates of chronic low back pain measured by arterial spin labeling. *Anesthesiology*.2011;115(2) : 10.1097/ALN.0b013e318220e880364-374 10.1097/ALN.0b013e318220e880 Reference source 21720241PMC3286828

[13] HowardMASandersDKrauseKO'MuircheartaighJFotopoulouAZelayaFThackerMMassatNHugginsJPVennartWChoyEDanielsMWilliamsSC: Alterations in resting-state regional cerebral blood flow demonstrate ongoing pain in osteoarthritis: An arterial spin-labeled magnetic resonance imaging study. *Arthritis Rheum.*.2012;64(12) : 10.1002/art.376853936-3946 10.1002/art.37685 Reference source 22933378

[14] LiuJDuMWangXZhangJManorBJiangXFangWWangD: Quantitative cerebral blood flow mapping and functional connectivity of postherpetic neuralgia pain: a perfusion fMRI study. *Pain*.2013;154(1) : 10.1016/j.pain.2012.09.016110-118 10.1016/j.pain.2012.09.016 Reference source 23140909

[15] LoggiaMLKimJGollubRLVangelMGKirschIKongJWasanADNapadowV: Default mode network connectivity encodes clinical pain: an arterial spin labeling study. *Pain*.2013;154(1) : 10.1016/j.pain.2012.07.02924-33 10.1016/j.pain.2012.07.029 Reference source PMC353495723111164

[16] YoussefAMGustinSMNashPGReevesJmPetersenETPeckCCMurrayGMHendersonLA: Differential brain activity in subjects with painful trigeminal neuropathy and painful temporomandibular disorder. *Pain*.2014;155(3) : doi:10.1016/j.pain.2013.11.008467-475 10.1016/j.pain.2013.11.008 Reference source 24269492

[17] KucyiADavisKD: The dynamic pain connectome. *Trends Neurosci*.2015;38(2) : 10.1016/j.tins.2014.11.006.86-95 10.1016/j.tins.2014.11.006. Reference source 25541287

[18] SpornsO: The human connectome: origins and challenges. *Neuroimage*.2013;38: 10.1016/j.neuroimage.2013.03.02353-61 10.1016/j.neuroimage.2013.03.023 Reference source 23528922
